# Comment on: Impaired health-related quality of life in idiopathic inflammatory myopathies: a cross-sectional analysis from the COVAD-2 e-survey

**DOI:** 10.1093/rap/rkae097

**Published:** 2024-08-14

**Authors:** Josef Finsterer

**Affiliations:** Neurology Department, Neurology & Neurophysiology Center, Vienna, Austria

**Keywords:** idiopathic inflammatory myositis, autoimmune rheumatic disease, quality of life, mental health, SARS-CoV-2

Dear Editor, We read with interest the article by Yoshida *et al*. [1] about a study on the quality of life in 1582 patients with idiopathic inflammatory myositis (IIM), 4700 patients with non-IIM autoimmune rheumatic disease (AIRD), 545 patients with non-rheumatic autoimmune disease (nrAID) and 2675 controls using the scores for global physical health (GPH) and global mental health (GMH) scores. The lowest GPH levels were found in IIM and non-IIM AIRD patients [1]. Mean GMH levels were lower in IIM patients than in controls [1]. The factors responsible for low GPH levels were inclusion body myositis, comorbidities, active disease and glucocorticoid use [1]. Predictive factors for low GMH scores were overlap myositis, interstitial lung disease, depression, active disease, lower PROMIS Physical Function 10a score and higher PROMIS Fatigue 4a score [1]. It was concluded that both physical and mental health are significantly impaired in IIM patients, especially in those with comorbidities and increased fatigue. This highlights the importance of patient-reported experiences and optimized multidisciplinary care to improve the well-being in people with IIMs [1]. The study is impressive, but several points require discussion.

The first point is that the study was based on an electronic questionnaire [1]. Electronic questionnaires have several disadvantages. First, it cannot be ensured that the addressee is actually the patient in question and not a relative, friend or carer who is completing the questionnaire, i.e. not the patient himself. Second, missing data cannot simply be completed if an addressee does not fill in all of the questions asked. Third, desirable new data can no longer be generated and added to the data set. Fourth, the information provided by the patient cannot be easily verified. We should know how many of the patients were excluded due to missing or incomplete data.

The second point is that assessing physical and mental health using an electronic questionnaire is inadequate because the questions are predetermined and do not allow for further questions or explanation. Physical health should be assessed through clinical and instrumental examinations by competent physicians. Assessing physical and mental health using GPH and GMH scores alone has the disadvantage that the reproducibility of certain findings cannot be verified.

The third point is that it is inacceptable that a significant number of controls had comorbidities such as asthma, liver disease, COPD, interstitial lung disease (ILD), cardiovascular disease, diabetes, hyperlipidaemia, stroke, epilepsy, tuberculosis, HIV, anxiety disorders, depression, insomnia, eating disorder, which can greatly influence HPG and HMG values. Therefore, control subjects with comorbidities should be excluded from the study in order not to distort the study results. Furthermore, it was not explained why pregnant and breast-feeding women were excluded from the study.

In summary, the excellent study has limitations that should be addressed before drawing final conclusions. Clarifying the weaknesses would strengthen the conclusions and could improve the study. The quality of life of patients with IIM, non-IIM AIRD, and nrAID can only be reliably assessed on site and compared with controls only if comorbidities affecting quality of life have been excluded.

## Data Availability

All data are available from the corresponding author.
